# Deep Convolutional Neural Network-Based Hemiplegic Gait Detection Using an Inertial Sensor Located Freely in a Pocket

**DOI:** 10.3390/s22051920

**Published:** 2022-03-01

**Authors:** Hangsik Shin

**Affiliations:** Department of Convergence Medicine, Asan Medical Center, University of Ulsan College of Medicine, 88, Olympic-ro 43-gil, Songpa-gu, Seoul 05505, Korea; hangsik.shin@gmail.com

**Keywords:** accelerometry, convolutional neural network, gait analysis, gyroscope, hemiplegia, inertial signal

## Abstract

In most previous studies, the acceleration sensor is attached to a fixed position for gait analysis. However, if it is aimed at daily use, wearing it in a fixed position may cause discomfort. In addition, since an acceleration sensor can be built into the smartphones that people always carry, it is more efficient to use such a sensor rather than wear a separate acceleration sensor. We aimed to distinguish between hemiplegic and normal walking by using the inertial signal measured by means of an acceleration sensor and a gyroscope. We used a machine learning model based on a convolutional neural network to classify hemiplegic gaits and used the acceleration and angular velocity signals obtained from a system freely located in the pocket as inputs without any pre-processing. The classification model structure and hyperparameters were optimized using Bayesian optimization method. We evaluated the performance of the developed model through a clinical trial, which included a walking test of 42 subjects (57.8 ± 13.8 years old, 165.1 ± 9.3 cm tall, weighing 66.3 ± 12.3 kg) including 21 hemiplegic patients. The optimized convolutional neural network model has a convolutional layer, with number of fully connected nodes of 1033, batch size of 77, learning rate of 0.001, and dropout rate of 0.48. The developed model showed an accuracy of 0.78, a precision of 0.80, a recall of 0.80, an area under the receiver operating characteristic curve of 0.80, and an area under the precision–recall curve of 0.84. We confirmed the possibility of distinguishing a hemiplegic gait by applying the convolutional neural network to the signal measured by a six-axis inertial sensor freely located in the pocket without additional pre-processing or feature extraction.

## 1. Introduction

Gait is an important index for evaluating musculoskeletal diseases and degenerative diseases that increase with aging. The number of studies on gait analysis for disease diagnosis and daily health management is continuously increasing. Among the various methods for gait analysis, gait analysis using an inertial sensor, such as a gyroscope or an accelerometer, has an advantage, since it can be implemented with only a commercially available small chip. Therefore, the system is simple and easier to use than is a gait analysis mat or smart insole. In addition, the most recent personal smart and wearable devices are equipped with an inertial sensor, so it is easy to perform gait analysis without an additional system. Inertial sensors are widely applied for gait analysis for purposes that range from detecting gait-specific features [1,2] and general gait events [3,4,5,6] to clinical applications in the rehabilitation training monitoring of hemiplegic patients [7,8,9,10,11] and patients with Parkinson’s disease [12,13] or Alzheimer’s disease [14].

In our previous study, we developed a wearable acceleration measurement system that was fixed to the waist to obtain gait data from normal and hemiplegic patients and classified normal gait and hemiplegic gait using a random-forest classifier based on 165 extracted features [7,10]. In most previous studies, the acceleration sensor was attached to a fixed position to acquire a signal [6,15,16], and gait features were detected for pattern classification. This approach is advantageous in securing high accuracy because it can maintain the same acceleration–sensor axial direction when acquiring a signal and can detect only the main characteristics of walking, so that the influence of factors other than walking can be reduced. However, if it is aimed at daily use, wearing it in a fixed position may cause discomfort. In addition, in modern society, since an acceleration sensor can be built into smartphones that people always carry, it is more efficient to use such a sensor rather than wear an additional acceleration sensor. Most of the previous studies that analyzed gait using an accelerometer analyzed gait using sensors attached to fixed positions such as the low back, neck, leg, and thigh in a fixed direction [7,17,18,19,20,21,22,23]. However, this method of use is difficult to apply in daily life because it constrains the body, and it is very different from the usage style of smartphones used in various and free ways, so it is difficult to be installed directly on a smartphone. Therefore, to improve usability in daily life, there is a need for a system capable of ensuring use in which the wearing method, or the wearing position or direction, is not fixed. As the algorithm of gait analysis, studies are being conducted to extract specific gait features of gait events using the acceleration sensor built into a smartphone [2,3,24,25]. However, when a smart phone is used, the pattern of use is different for each user. Thus, the signal acquired for each axis of the acceleration sensor may differ, requiring corrections. In addition, when a feature detected from an original signal is used as an input to a classifier, the complexity of the system that results from the feature extraction process may increase. Furthermore, the classifier input may be distorted because of an error generated in the feature extraction process, and the error may be propagated if the distorted input leads to incorrect output. Therefore, the development of a method that can distinguish gait features by an analysis that uses the original signal as is without feature extraction can improve the reliability of the system.

Deep learning-based methods do not require additional feature extraction or generation, because the model generates features on its own from data and classifies them using the generated features. In addition, since it is easy to apply to a complex nonlinear classification problem or to expand the input dimension, it can be used as an appropriate way to analyze signals from multiple input channels, such as from an acceleration sensor. In recent gait analysis studies, an increasing number of cases have applied machine learning to analyze the human gait including pathological gait [26,27,28,29]. For example, the deep learning method is applying in analysis of kinetic patterns of gait [28,29,30,31] or to detect health abnormalities [32] and abnormal gaits [33], pathological gait of Huntington disease [26], or post-stroke gait [27]. Studies analyzing the signals acquired from accelerometers by machine learning are also being continuously published, and studies have been conducted to detect pathological gaits such as those in patients with multiple sclerosis [34] and Parkinson’s disease [12,13] through wearable sensors and machine learning.

The purpose of this study was to detect hemiplegic gait using an accelerometer located freely in a pocket without fixing the position and orientation, classify hemiplegic gait using the explainable artificial intelligence (AI), Grad-Cam, and identify which features of the measured signal waveforms characterized hemiplegic gait. To this end, we acquired data by conducting clinical trials on hemiplegic patients and normal people and developed and validated a deep learning model for classifying gait without additional pre-processing such as feature extraction.

## 2. Materials and Methods

### 2.1. Data Acquisition System

We measured acceleration and angular velocity using a measurement system consisting of a six-axis inertial sensor, a microcontroller, and a Bluetooth module (MPU9250, InvenSense, San Jose, CA, USA) with a built-in three-axis accelerometer and a three-axis gyroscope as the inertial sensor, which measures linear acceleration during walking and changes in angular velocity caused by rotation. We used an ultra-low-power microcontroller (MPS430G2553, Texas Instruments, Dallas, TX, USA) [35] that collected data from the inertial sensor and transmitted it wirelessly to a PC through a Bluetooth version 2.1 module (FB155BC, Firmtech, Sungnam, Korea). We designed the system to be 43 mm wide and 33 mm long, with a maximum thickness of 8.6 mm. The developed system recorded signals on 6 channels simultaneously with three-axis acceleration and three-axis angular velocity. We set the sampling rate for each channel to 125 Hz.

### 2.2. Experimental Protocol

We conducted this study after obtaining Institutional Review Board (IRB) approval (IRB no. SCH2016-130) from Suncheon St. Carollo Hospital. We conducted the experiment on participants who voluntarily agreed and signed the consent form. The subjects in this study included a normal group and a group of hemiplegic patients. We selected the normal group from the volunteers who obtained a normal grade (5 points) in all measurement categories of a manual muscle test (MMT) conducted by a therapist from the rehabilitation medicine team. We selected the group of hemiplegic patients from among adult patients who were diagnosed with a stroke, were undergoing rehabilitation treatment, had no orthopedic disease, had a clear level of consciousness, and could walk independently for more than 20 m on a level surface without the aid of an assistive device. We performed the experiment as a 20-m round-trip walking test in a 20-m-long corridor with no obstacles, recorded one walking signal for each one-way trip, acquired two records per study subject, and recorded movements during walking using the developed system. At this time, the measurement system recorded the signal while it was in the pocket of the study subject without specifying the direction or location. The experiment was conducted according to the following procedure.

1. The test subject randomly put the measurement system in the pocket of a jacket and waited at the starting point.
2. When the start signal was given after the PC application and measurement system were connected, the test subject walked along the 20-m-long corridor at their usual walking speed.
3. After walking 20 m, the subject turned around and waited.
4. When the start signal was given, the subject returned along the 20-m-long corridor.
5. The subject waited at the starting point.
6. We ended the experiment by terminating the application and saving the measurement data.

### 2.3. Machine Learning Model

We used a machine learning model based on a convolutional neural network (CNN) [36] to classify hemiplegic gaits. CNN is a neural network that can be used to derive results from a convolution operation using a multidimensional kernel. It has the advantage of using features that reflect various dimensional features of the data. CNN is known to have a good performance when input samples have spatio-temporal connectivity, so it has been frequently used in gait analysis studies. In previous gait analysis studies, CNN has used for gait recognition [37,38,39], gait acceleration classification [40], gait event detection [41], or gait authentication and labeling [42]. Our CNN model consisted of multiple convolutional layers followed by a max pooling layer and a fully connected layer. The input layer of the proposed model was 625 × 6, which reflects the number of samples in a 5 s segment and 6 axes. The first convolution layer contained 32 kernels that were 16 × 2 with stride 1. The second convolution layer contained 64 kernels that were 8 × 2 with stride 1. The third convolution layer contained 128 kernels that were 4 × 2 with stride 1. The fourth and subsequent convolutional layers are designed with the same structure; they contained 256 kernels that were 2 × 2 with stride 1. Max pooling layers had kernels that were 2 × 2 with stride 2. The convolutional layer used ReLU as an activation function and adapted batch normalization after activation. The output of the last max pooling layer went into a series of a fully connected layer by means of flattening. The fully connected layer included dropout [43], and ReLU was applied as an activation function, and the output of fully connected layer was fed into a Softmax layer with two class labels. We used the adaptive moment estimate (Adam) [44] for cost optimization in model development with a 0.9 exponential decay rate for the moving average gradient (${\_}_{1}$) and a 0.999 exponential decay rate for the moving average of the squared gradient (${\_}_{2})$. We performed Bayesian optimization [45] to optimize the number of convolutional layers, dropout rate, and learning rate. Bayesian optimization was performed for the number of convolutional layers of layers (1 to 6), dropout rate of (0 to 0.5), number of nodes for dense layer of (256 to 2048), batch size of (1 to 100), and learning rate of (0.001 to 0.01). We developed and validated the proposed CNN model with a 3.8 GHz Intel Core i7-8700 processor, 64 GB 1600 MHz DDR3 RAM, NVIDIA Geforce GTX 3090, Python 3.6.7, and Tensorflow 2.0. The input data were generated by stacking the six-channel signals acquired from the three-axis acceleration and three-axis gyroscope in the form of a 2D image. Since the input signal was generated based on 5 s of data, it had 625 data samples × 6 channels sampled at 125 Hz for 5 s.

### 2.4. Leave-One-Out Cross Validation

**Figure 1:**
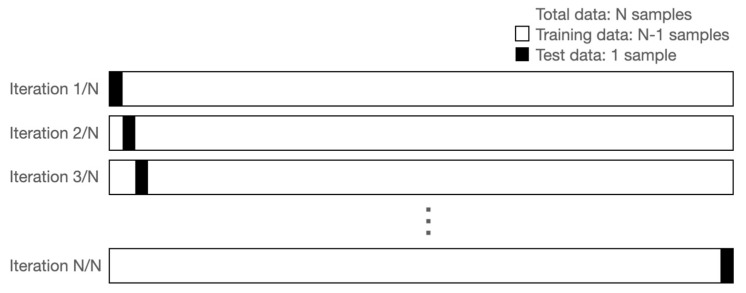
Leave-one-out cross validation.

The leave-one-out CV (LOOCV) method generates as many N models as there are samples and calculates the test set performance with samples excluding one sample when making each model (Figure 1). The final performance is obtained by averaging the performance of N tests. The advantage of LOOCV is that there is no randomness, because all samples are tested one at a time. Unlike the validation set approach, very stable results can be obtained. In addition, since only one sample is used as a test set, a model can be created using a large amount of training data. When verifying the LOOCV, we used 20% of the training set as a validation set by random sampling. When selecting the validation set, we used stratified sampling so that the normal and hemiplegic data had the same distribution as the training set. In dividing the fold, the training set and test set were divided into subjects. For example, if the data of the 1st to the (N-1)th subjects out of a total of N volunteers were used as the training set, the data of the Nth subject were used as the test set. Learning was performed up to the epoch where the validation loss was minimal, and the performance was evaluated by applying the test set. We used the *EarlyStopping* and *Modelcheckpoint* options of Tensorflow to find the model with minimal validation loss. 

### 2.5. Statistical Analysis

The validation process was repeated N times, with each of the N-1 sub-datasets used exactly once as the validation data. The results from the folds were averaged to produce a single estimation. The accuracy (Ac), precision (Pr), and recall (Re) of the results were statistically analyzed. Here, Ac is a probability of a correct decision in every class, which can be interpreted in the same sense as the probability of detecting of both normal and hemiplegic gaits. Pr is the proportion of what the model classifies as hemiplegic gait classified by the model that was correct. Recall is the proportion of what the model predicted as hemiplegic gait among those that were actually hemiplegic gait. The Ac, Pr, and Re equations are presented in Equations (1)–(3), where true positive (TP) is the number of cases where an actual hemiplegic gait was predicted by the classification model as a hemiplegic gait, true negative (TN) was the number of cases in which an actual normal gait was predicted by the classification model as a normal gait, false positive (FP) was the number of cases where the classification model predicted an actual normal gait as a hemiplegic gait, and false negative (FN) was the number of cases where the classification model predicted an actual hemiplegic gait as a normal gait.

(1) $$\;\mathrm{Accuracy}\;\left(\mathrm{Ac}\right)=\frac{\mathrm{TP}+\mathrm{TN}}{\mathrm{TP}+\mathrm{FP}+\mathrm{TN}+\mathrm{FN}}\times 100$$

(2) $$\;\mathrm{Precision}\;\left(\mathrm{Pr}\right)=\frac{\mathrm{TP}}{\mathrm{TP}+\mathrm{FP}}\times 100$$

(3) Recall (Re)=TPTP+FN×100

To evaluate overall performance, we derived the receiver operating characteristics (ROC) curve and precision-recall curve. The area under the receiver operating characteristic (AUROC) is a performance metric for evaluating binary classification models. The AUROC is calculated as the area under the ROC curve. An ROC curve shows the trade-off between the true positive rate (TPR) and the false positive rate (FPR) across different decision thresholds. The precision-recall curve shows the trade-off between the true positive rate and the positive predictive value for a predictive model using different probability thresholds. The area under the precision-recall curve (AUPRC) is also a useful performance metric for imbalanced data in a problem setting where finding positive examples is important. 

### 2.6. Grad-Cam

A class activation map (CAM) is a way to find the main elements that have a great influence on the classification result in the image by putting global average pooling GAP) into the last layer of the CNN model [46]. CAM is a type of AI that can explain the inner workings of a machine learning model that is considered a black box and can tell which element of the input data is important information for classification. However, to apply CAM, a GAP layer must be added to the last layer of the model, so there is a limitation when constructing a deep learning model. Grad-Cam is a way of calculating weights that are connected to features by gradients. Thus, CAM can be obtained without modifying the original machine learning model structure [47]. Since Grad-CAM can use the existing model structure as is, it can be applied to most CNN models, such as those that include a fully connected layer, a structured output, or multi-modal input.

### 2.7. Uncertainty

Uncertainty in artificial intelligence can be defined as “the possibility of making erroneous judgments due to the lack of appropriate information necessary for judgment or decision-making”. For example, since there is no expression of ‘don’t know’ in the output of deep learning, when new data that has never been learned is input, the result can be expressed as if it were a normal prediction even though it is a completely wrong result. There is a way to use the Bayesian model to obtain the uncertainty, but it is difficult to apply in practice because the amount of computation is huge. Yarin Gal proposed a method to obtain uncertainty by approximating the neural network model to a Bayesian model through dropout [48]. A neural network to which Dropout is applied does not perform learning on all layers of the neural network, but rather performs learning through a neural network in which some neurons in the input layer or hidden layer in the neural network are omitted. In this case, various neural networks with different weights are generated according to the combination of selected neurons, and the uncertainty of the model can be evaluated by comparing the results of these neural networks. Therefore, in this study, uncertainty was calculated by adjusting the Dropout of the fully connected layer in the range of 0–0.5.

## 3. Results

### 3.1. Dataset

**Figure 2:**
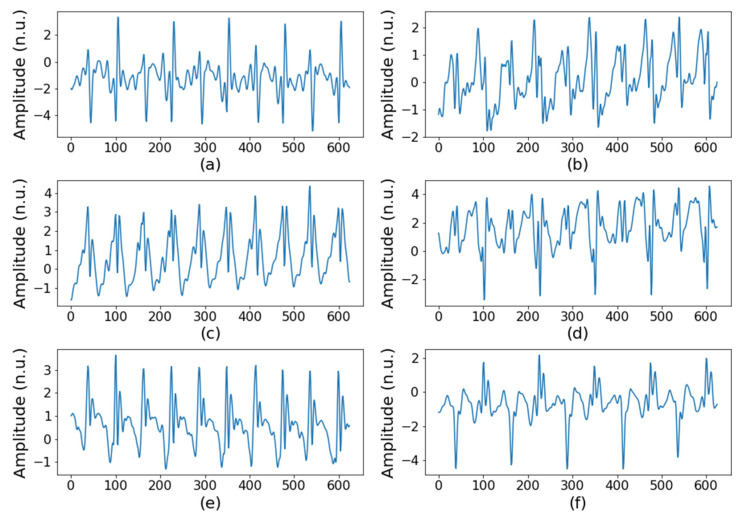
Inertial signals of normal gait: (**a**) acceleration in the *x* axis, (**b**) angular velocity in the *x* axis, (**c**) acceleration in the *y* axis, (**d**) angular velocity in the *y* axis, (**e**) acceleration in the *z* axis, and (**f**) angular velocity in the *z* axis.

**Figure 3:**
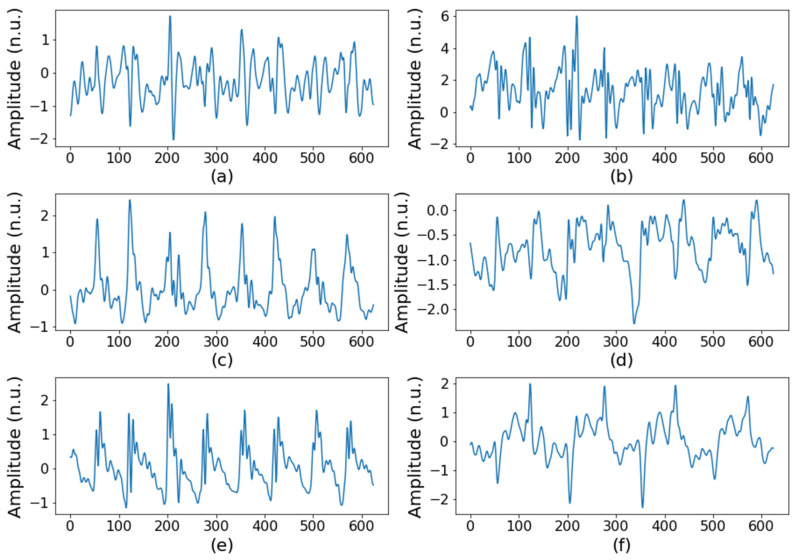
Inertial signals of hemiplegic gait: (**a**) acceleration in the x axis, (**b**) angular velocity in the x axis, (**c**) acceleration in the y axis, (**d**) angular velocity in the y axis, (**e**) acceleration in the z axis, and (**f**) angular velocity in the z axis.

In total, 42 volunteers, including 21 hemiplegic patients, participated in the clinical trial. The demographic information of the clinical trial participants is shown in Table 1. Differences between the sexes and the ages of normal and hemiplegia group was not statistically significant in a Chi-square test (*p* > 0.05). For testing significant difference in age groups, the Chi-square test was conducted to the frequency of ages grouped by each decade. Among the hemiplegic patients, 7 were left paralyzed, 11 were right paralyzed, and 3 had bilateral paralysis. Using the 20-m round-trip walking test, we acquired two datasets per study subject and thus collected 84 datasets, of which 42 displayed normal walking and 42 displayed hemiplegic walking. After that, to create a dataset as input for the machine learning model, we extracted a walking segment of a specific length from each person’s walking data. The input data were generated by repeatedly extracting a random 5 s section from the walking signal for data augmentation. Therefore, 10 segments for one-way walking and 20 segments from round-trip walking for each subject were extracted. As a result, 840 segments from a total of 42 subjects were used as input data. In performing LOOCV, 8 subjects’ data (20% of 41 training subjects) were used as the validation set; as a result, per each fold, 33 participants’ data (600 segments), 8 participants’ data (160 segments), and a participant’s data (20 segments) were used for training, validation, and testing, respectively. Each segment was composed of six-axis signals, including three-axis acceleration and three-axis angular velocity signals. Figure 2 and Figure 3 show an example of an acceleration signal acquired while walking. Figure 2 is a normal gait, and Figure 3 shows that the hemiplegic patient’s gait was less regular than is the normal gait.

### 3.2. CNN Model

**Figure 4:**
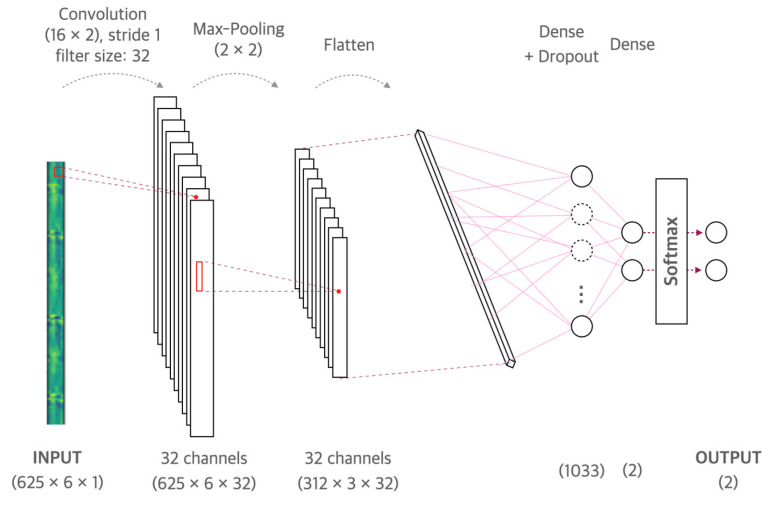
Model based on a convolutional neural network for gait classification.

As a result of optimization, the proposed model was confirmed to have the best performance in the CNN architecture of one layer, with number of fully connected nodes of 1033, batch size of 77, learning rate of 0.001, and dropout rate of 0.48. Figure 4 shows the structure of the CNN model optimized through Bayesian optimization.

### 3.3. Classification Results

**Figure 5:**
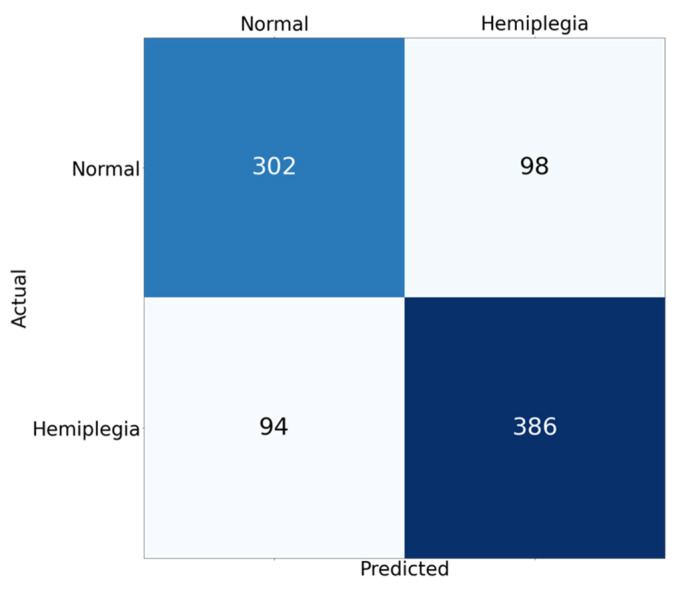
Confusion matrix of the result of proposed classification model. The numbers of true positives, true negatives, false positives, and false negatives are 386, 302, 98, and 94, respectively.

**Figure 6:**
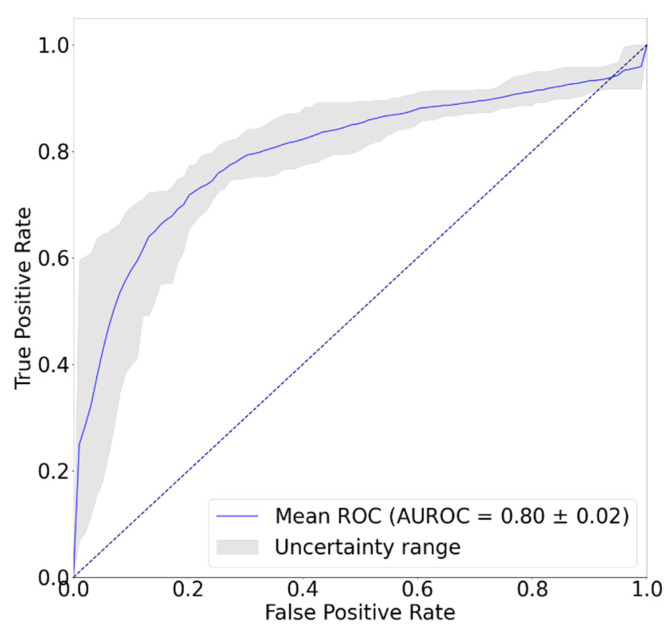
Receiver operating characteristic (ROC) curve of proposed model. Grey area represents the range of uncertainty. AUROC: Area under curve of ROC curve.

**Figure 7:**
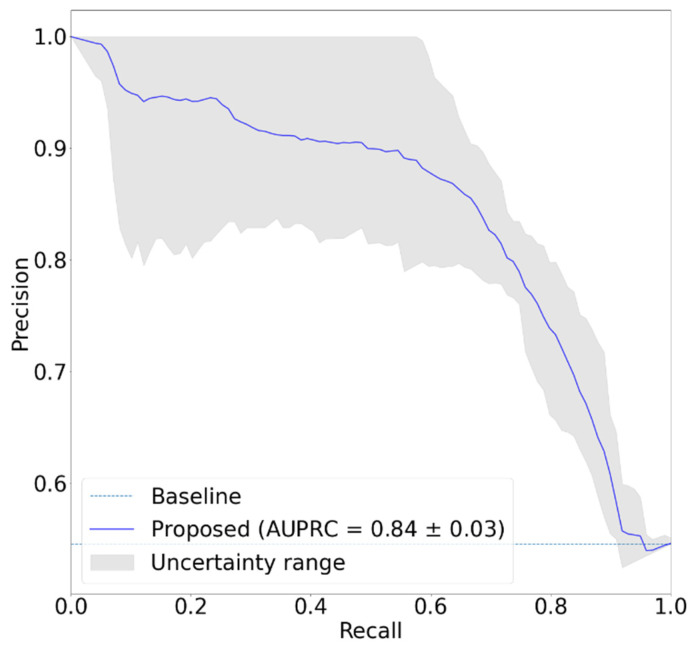
Precision-recall curve of proposed model. Grey area represents the range of uncertainty. AUPRC: Area under curve of precision-recall curve.

**Figure 8:**
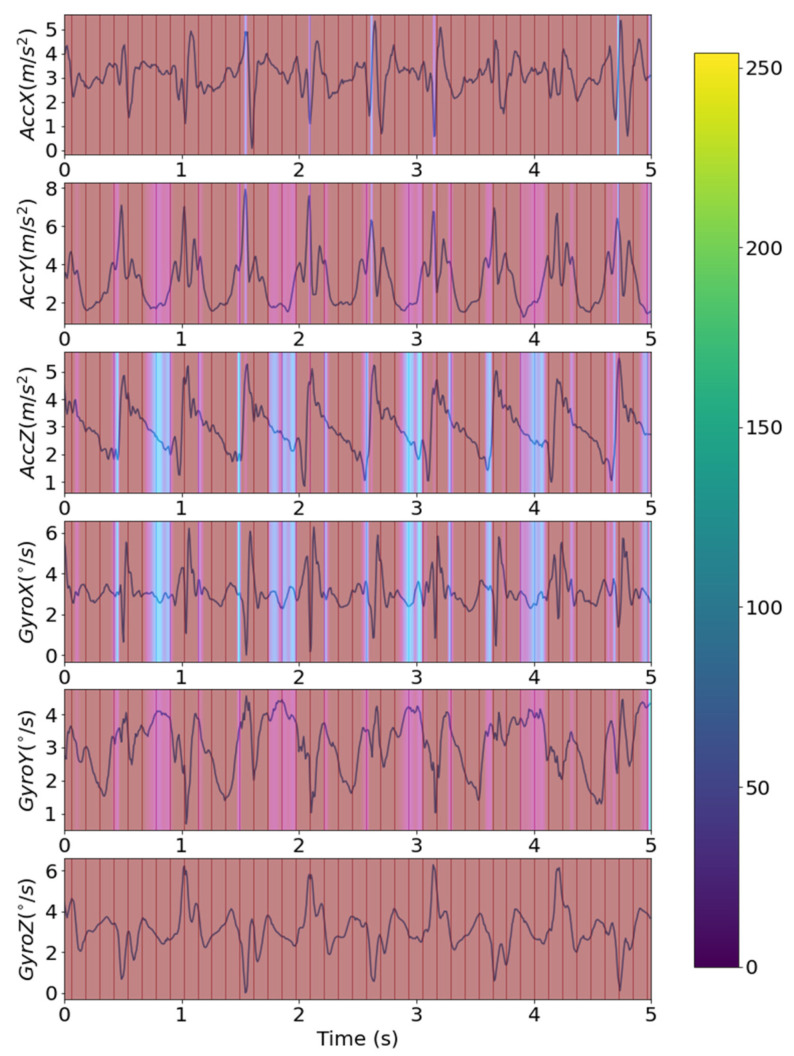
Example of heatmap using Grad-Cam.

Figure 5 shows the confusion matrix of the classification result. Total number of samples was 880 from 88 records by augmenting the data sample using random interval sampling. In Figure 5, since hemiplegia is annotated as ‘positive’, the number of true positives is 386, and 302, 98, and 94 correspond to the true negatives, the false positives, and the false negatives, respectively. Table 2 shows the results of hemiplegic gait classification using the proposed model. The accuracy, precision, recall, and F1 scores of the proposed model were 0.78, 0.80, 0.80, and 0.80, respectively, and the AUC was 0.80. Considering that an 0.8 AUC is the criterion for reasonable performance in most studies, this result suggests that the proposed model worked well for detecting unilateral walking. Figure 6 shows the ROC curve of the classification model. The blue line shows the identical line, and the dark-orange line indicates the ROC curve of the developed model. In Figure 6, the ROC curve is in the upper left corner and shows that the model worked well. Figure 7 shows the precision-recall curve of the developed model. The AUPRC of the precision-recall curve was 0.84. In Figure 6 and Figure 7, the grey area represents the range of uncertainty derived by changing dropout rate in range of 0–0.5. In the AUROC, it was confirmed that the uncertainty was within 5% of the average AUC in the 0.78–0.82 range, and in the AUPRC, the uncertainty was within 3.5% of the AUPRC in the 0.81–0.87 range. Figure 8 is an example of confirming with Grad-Cam, where the characteristics of the input signal influenced the classification results. In this study, since the measuring device was positioned freely in the pocket, the characteristics of each axis may differ depending upon the direction in which the measuring device was located. Therefore, rather than analyzing the characteristics of a specific axis, one needs to analyze the results according to the shape of the overall signal. A qualitative analysis using a heatmap confirmed that most of the large and rapid changes contributed more than the slow changes of waveform.

## 4. Discussion

We proposed a CNN-based machine learning model that distinguishes hemiplegic walking from normal walking using acceleration and angular velocity signals measured freely in a pocket without being fixed at a specific position. The developed model had an accuracy of 0.78, an AUROC of 0.80, and an AUPRC of 0.84. In many existing gait analysis studies, gait characteristics were detected using data acquired from sensors attached at a specific position and direction, and gait was classified based on the detected characteristics. In the case of an inertial sensor, since it has directionality, when the wearing position or direction is fixed, a signal having a consistent pattern can be obtained according to a specific motion; thus, it has the advantage of easy analysis. Therefore, many previous studies have analyzed gait using a sensor with a fixed position and direction, but it has not been popularized due to the inconvenience of usage and wearing method. In this study, we measured signals and analyzed the type of gait using devices that had no positional or directional constraints. This was similar to measuring acceleration and angular velocity signals while walking using a personal mobile phone equipped with an acceleration sensor or gyroscope as a default. Hence, the developed algorithm is easy to apply to personal mobile devices. Therefore, the algorithm developed in this study is easy to apply to existing mobile devices, and it can be used to always estimate the degree of improvement in hemiplegia signs or symptoms through daily gait monitoring using smartphones. Especially, we used three-axis acceleration and three-axis angular velocity raw signals, which did not undergo additional feature extraction processes, as inputs for the machine learning model. Most of the existing studies classified gait using features derived by means of signal processing, which requires a complex operation for feature detection, and an error that occurs in the feature detection process may affect the result analysis. Thus, there is a disadvantage, in that the gait classification accuracy depends upon the feature extractor performance. In contrast, using the original signal proposed in this study as an input did not require a separate feature extraction process when using a deep learning model, so it was possible to analyze the gait more robustly to input changes. The machine learning model we proposed in this study showed a reasonable performance, even though there was no additional preprocessing or feature detection, because the proposed CNN model preserves the spatiotemporal correlation of multi-channel data.

In addition, in this study, Grad-Cam was used as an explainable AI to characterize the waveforms with a major influence on the classification of hemiplegia. Since the axis direction was set randomly, it is difficult to generalize the results. However, the Grad-Cam results suggest that the early-stage gait signal, which showed a sharp waveform change in the axial signal where gait was clearly distinguished, had a relatively greater effect on gait classification than the late-stage gait signal. The above results showed the possibility that explainable AI can be useful in analyzing or classifying the mechanism of an abnormal gait. Previous studies on hemiplegic gait detection using inertial sensors were mostly performed by classifying gait based on inertial sensors fixed in specific directions at specific locations on the human body. In a study by Christou et al., six time and frequency domain features were extracted from signals collected from seven inertial sensors attached to four body sites, and hemiplegic gait was classified with an accuracy of 87.7% using a neural network [18]. In another study, hemiplegic gait was classified with 81.9% test set accuracy through transfer learning with inertial sensors attached to both lower limbs [22]. In addition, a study analyzing gait using a sensor fixedly worn behind the waist belt and along the spinal cord showed that LSTM-CNN could classify hemiplegic gait with up to 93.1% test set accuracy [33]. The above studies were similar to our study in that they used an inertial sensor to classify hemiplegia, but they are all different from our study in that they acquired signals from an inertial sensor attached to a specific location and in a specific direction. In addition, since some studies [22,33] were based on simulated hemiplegic data from normal people, not actual hemiplegic patients, it is difficult to directly compare the results to those of our study. The results of this study were derived from data collected from actual hemiplegic patients and showed accuracy similar to that in a previous study [18] performed on hemiplegic patients. These results suggest that the method proposed in this study could improve convenience without significant degradation in accuracy. In addition, the proposed technology should be applied to devices with built-in acceleration sensors such as smartphones. When applied to personal portable devices, it can be used for monitoring the occurrence of hemiplegia and increasing or decreasing the severity of hemiplegia during daily life.

However, this study has some limitations and most of them are stemmed from small subject size. In this study, we classified the presence or absence of hemiplegia with high accuracy using the proposed model, but a larger group of subjects is required because having only 21 subjects in each group could not provide a normal distribution. In addition, we did not address the severity of paralysis or hemiplegia because a large amount of data is required to develop a deep learning model, and we had only 42 subjects for this study, which was insufficient to enable analysis by paralysis and severity. Since a model created with a small number of study subjects may have biased characteristics, model improvement using more subject data is required to minimize overfitting. In addition, although not performed in this study, external validation using a data set obtained separately from the data set used for development should be performed to confirm the validity of the developed model.

## 5. Conclusions

In this study, we confirmed the possibility of distinguishing hemiplegic gaits by means of a deep learning model without additional pre-processing or feature extraction using a six-axis inertial signal measured at random locations in a pocket. Moreover, we investigated the effective features of the inertial signal waveforms using the explainable AI, Grad-Cam. The result of proposed method shows the reasonable performance (0.8 AUROC) in hemiplegia classification. However, this study on a nonparametric group needs to be verified in a more extensive study. In addition, our results could be more generalizable by studies including subjects of various sexes, ages, hemiplegia axes, and hemiplegia severity. The system can be installed on smartphones in the future and applied to the early diagnosis and prognosis of hemiplegia patients.

## Figures and Tables

**Table 1:** Demographic information for participants.

Parameters.	Normal (*N* = 21)	Hemiplegia (*N* = 21)
Age (years)	53.0 ± 16.0 (range: 27–77)	62.6 ± 9.2 (47–78)
Sex (M/F, *N*)	11/10	13/8
Height (cm)	165.0 ± 7.6 (150–178)	165.1 ± 11.0 (145–178)
Weight (kg)	66.3 ± 9.9 (48–82)	66.2 ± 14.6 (38–90)
Paralyzed side (N)	not applicable	Left (7), Right (11), Both (3)

**Table 2:** Hemiplegic gait classification performance of the proposed model (*N* = 84). AUROC: area under of receiver operating characteristic curve; AUPRC: area under of the precision-recall curve.

Evaluation Metrics	Values
Accuracy	0.78
Precision	0.80
Recall	0.80
F1 score	0.80
AUROC	0.80
AUPRC	0.84

## References

[1] Aminian K., Rezakhanlou K., De Andres E., Fritsch C., Leyvraz P.-F., Robert P. (1999). Temporal feature estimation during walking using miniature accelerometers: An analysis of gait improvement after hip arthroplasty. Med. Biol. Eng. Comput..

[2] Nishiguchi S., Yamada M., Nagai K., Mori S., Kajiwara Y., Sonoda T., Yoshimura K., Yoshitomi H., Ito H., Okamoto K. (2012). Reliability and validity of gait analysis by android-based smartphone. Telemed. E-Health.

[3] Thang H.M., Viet V.Q., Thuc N.D., Choi D. Gait identification using accelerometer on mobile phone. Proceedings of the 2012 International Conference on Control, Automation and Information Sciences (ICCAIS).

[4] Lee J.-A., Cho S.-H., Lee J.-W., Lee K.-H., Yang H.-K. Wearable accelerometer system for measuring the temporal parameters of gait. Proceedings of the 2007 29th Annual International Conference of the IEEE Engineering in Medicine and Biology Society.

[5] Bugané F., Benedetti M., Casadio G., Attala S., Biagi F., Manca M., Leardini A. (2012). Estimation of spatial-temporal gait parameters in level walking based on a single accelerometer: Validation on normal subjects by standard gait analysis. Comput. Methods Programs Biomed..

[6] Hartmann A., Luzi S., Murer K., de Bie R.A., de Bruin E.D. (2009). Concurrent validity of a trunk tri-axial accelerometer system for gait analysis in older adults. Gait Posture.

[7] Lee J., Park S., Shin H. (2018). Detection of hemiplegic walking using a wearable inertia sensing device. Sensors.

[8] Mizuike C., Ohgi S., Morita S. (2009). Analysis of stroke patient walking dynamics using a tri-axial accelerometer. Gait Posture.

[9] Moore S.A., Hickey A., Lord S., Del Din S., Godfrey A., Rochester L. (2017). Comprehensive measurement of stroke gait characteristics with a single accelerometer in the laboratory and community: A feasibility, validity and reliability study. J. Neuroeng. Rehabil..

[10] Park S., Lee J.S., Kwak J., Shin H. Design of the wearable device for hemiplegic gait detection using an accelerometer and a gyroscope. Proceedings of the 2017 39th Annual International Conference of the IEEE Engineering in Medicine and Biology Society (EMBC).

[11] Sekine M., Abe Y., Sekimoto M., Higashi Y., Fujimoto T., Tamura T., Fukui Y. Assessment of gait parameter in hemiplegic patients by accelerometry. Proceedings of the 22nd Annual International Conference of the IEEE Engineering in Medicine and Biology Society (Cat. No. 00CH37143).

[12] Rastegari E., Azizian S., Ali H. Machine learning and similarity network approaches to support automatic classification of parkinson’s diseases using accelerometer-based gait analysis. Proceedings of the 52nd Hawaii International Conference on System Sciences.

[13] Abdulhay E., Arunkumar N., Narasimhan K., Vellaiappan E., Venkatraman V. (2018). Gait and tremor investigation using machine learning techniques for the diagnosis of Parkinson disease. Future Gener. Comput. Syst..

[14] Chung P.-C., Hsu Y.-L., Wang C.-Y., Lin C.-W., Wang J.-S., Pai M.-C. Gait analysis for patients with Alzheimer’s disease using a triaxial accelerometer. Proceedings of the 2012 IEEE International Symposium on Circuits and Systems (ISCAS).

[15] Henriksen M., Lund H., Moe-Nilssen R., Bliddal H., Danneskiod-Samsøe B. (2004). Test–retest reliability of trunk accelerometric gait analysis. Gait Posture.

[16] Rispens S.M., Pijnappels M., van Schooten K.S., Beek P.J., Daffertshofer A., van Dieën J.H. (2014). Consistency of gait characteristics as determined from acceleration data collected at different trunk locations. Gait Posture.

[17] LeMoyne R., Mastroianni T. (2021). Implementation of Machine Learning Classification Regarding Hemiplegic Gait Using an Assortment of Machine Learning Algorithms with Quantification from Conformal Wearable and Wireless Inertial Sensor System. J. Biomed. Sci. Eng..

[18] Christou V., Arjmand A., Dimopoulos D., Varvarousis D., Tzallas A.T., Gogos C., Tsipouras M.G., Ploumis A., Giannakeas N. Neural Network-Based approach for Hemiplegia Detection via Accelerometer Signals. Proceedings of the 2021 6th South-East Europe Design Automation, Computer Engineering, Computer Networks and Social Media Conference (SEEDA-CECNSM).

[19] Lemoyne R., Mastroianni T. Implementation of a smartphone as a wearable and wireless gyroscope platform for machine learning classification of hemiplegic gait through a multilayer perceptron neural network. Proceedings of the 2018 17th IEEE International Conference on Machine Learning and Applications (ICMLA).

[20] Buckley C., Micó-Amigo M.E., Dunne-Willows M., Godfrey A., Hickey A., Lord S., Rochester L., Del Din S., Moore S.A. (2020). Gait asymmetry post-stroke: Determining valid and reliable methods using a single accelerometer located on the trunk. Sensors.

[21] Nakatsuchi T., Ikebuchi M., Nishikawa T., Sugahara T., Nakajima S., Morimoto M., Nakamura H. (2020). Analysis of Gait in Stroke Patients with Hemiplegia Using a Wearable Accelerometer. Osaka City Med. J..

[22] Pandit T., Nahane H., Lade D., Rao V. Abnormal Gait Detection by Classifying Inertial Sensor Data using Transfer Learning. Proceedings of the 2019 18th IEEE International Conference On Machine Learning And Applications (ICMLA).

[23] Zhang W., Smuck M., Legault C., Ith M.A., Muaremi A., Aminian K. (2018). Gait symmetry assessment with a low back 3D accelerometer in post-stroke patients. Sensors.

[24] LeMoyne R., Mastroianni T. (2017). Wearable and wireless gait analysis platforms: Smartphones and portable media devices. Wireless MEMS Networks and Applications.

[25] Iso T., Yamazaki K. Gait analyzer based on a cell phone with a single three-axis accelerometer. Proceedings of the 8th Conference on Human-Computer Interaction with Mobile Devices and Services.

[26] Zhang S., Poon S.K., Vuong K., Sneddon A., Loy C.T. (2019). A deep learning-based approach for gait analysis in Huntington disease. MEDINFO 2019: Health and Wellbeing e-Networks for All.

[27] Moro M., Marchesi G., Odone F., Casadio M. Markerless gait analysis in stroke survivors based on computer vision and deep learning: A pilot study. Proceedings of the 35th Annual ACM Symposium on Applied Computing.

[28] Horst F., Lapuschkin S., Samek W., Müller K.-R., Schöllhorn W.I. (2019). Explaining the unique nature of individual gait patterns with deep learning. Sci. Rep..

[29] Alharthi A.S., Yunas S.U., Ozanyan K.B. (2019). Deep learning for monitoring of human gait: A review. IEEE Sens. J..

[30] Begg R., Kamruzzaman J. (2005). A machine learning approach for automated recognition of movement patterns using basic, kinetic and kinematic gait data. J. Biomech..

[31] Su B., Smith C., Gutierrez Farewik E. (2020). Gait phase recognition using deep convolutional neural network with inertial measurement units. Biosensors.

[32] Pogorelc B., Bosnić Z., Gams M. (2012). Automatic recognition of gait-related health problems in the elderly using machine learning. Multimed. Tools Appl..

[33] Gao J., Gu P., Ren Q., Zhang J., Song X. (2019). Abnormal gait recognition algorithm based on LSTM-CNN fusion network. IEEE Access.

[34] McGinnis R.S., Mahadevan N., Moon Y., Seagers K., Sheth N., Wright J.A., DiCristofaro S., Silva I., Jortberg E., Ceruolo M. (2017). A machine learning approach for gait speed estimation using skin-mounted wearable sensors: From healthy controls to individuals with multiple sclerosis. PLoS ONE.

[35] Invensense T. MPU-9250 Datasheet. https://invensense.tdk.com/download-pdf/mpu-9250-datasheet/.

[36] O’Shea K., Nash R. (2015). An introduction to convolutional neural networks. arXiv.

[37] Lee S.-M., Yoon S.M., Cho H. Human activity recognition from accelerometer data using Convolutional Neural Network. Proceedings of the 2017 IEEE International Conference on Big Data and Smart Computing (Bigcomp).

[38] Dehzangi O., Taherisadr M., ChangalVala R. (2017). IMU-based gait recognition using convolutional neural networks and multi-sensor fusion. Sensors.

[39] Gadaleta M., Rossi M. (2018). Idnet: Smartphone-based gait recognition with convolutional neural networks. Pattern Recognit..

[40] Kreuter D., Takahashi H., Omae Y., Akiduki T., Zhang Z. (2020). Classification of human gait acceleration data using convolutional neural networks. Int. J. Innov. Comput. Inf. Control.

[41] Tian J. (2022). Adversarial vulnerability of deep neural network-based gait event detection: A comparative study using accelerometer-based data. Biomed. Signal Process. Control.

[42] Zhao Y., Zhou S. (2017). Wearable device-based gait recognition using angle embedded gait dynamic images and a convolutional neural network. Sensors.

[43] Srivastava N., Hinton G., Krizhevsky A., Sutskever I., Salakhutdinov R. (2014). Dropout: A simple way to prevent neural networks from overfitting. J. Mach. Learn. Res..

[44] Kingma D.P., Ba J. (2014). Adam: A method for stochastic optimization. arXiv.

[45] Snoek J., Larochelle H., Adams R.P. (2012). Practical bayesian optimization of machine learning algorithms. Adv. Neural Inf. Process. Syst..

[46] Zhou B., Khosla A., Lapedriza A., Oliva A., Torralba A. Learning deep features for discriminative localization. Proceedings of the IEEE Conference on Computer Vision and Pattern Recognition.

[47] Selvaraju R.R., Cogswell M., Das A., Vedantam R., Parikh D., Batra D. Grad-cam: Visual explanations from deep networks via gradient-based localization. Proceedings of the IEEE International Conference on Computer Vision.

[48] Gal Y., Ghahramani Z. Dropout as a bayesian approximation: Representing model uncertainty in deep learning. Proceedings of the 33rd International Conference on Machine Learning.

